# Supplementary material for: Exploring the Water–Soil–Crop Dynamic Process and Water Use Efficiency of Typical Irrigation Units in the Agro-Pastoral Ecotone of Northern China
Source: Plants (Basel). 2024 Jul 11;13(14):1916. doi: 10.3390/plants13141916 (PMC11280002; doi:10.3390/plants13141916)
Supplement: Supplementary file 1 [file plants-13-01916-s001.zip › plants-3016474-supplementary.pdf]

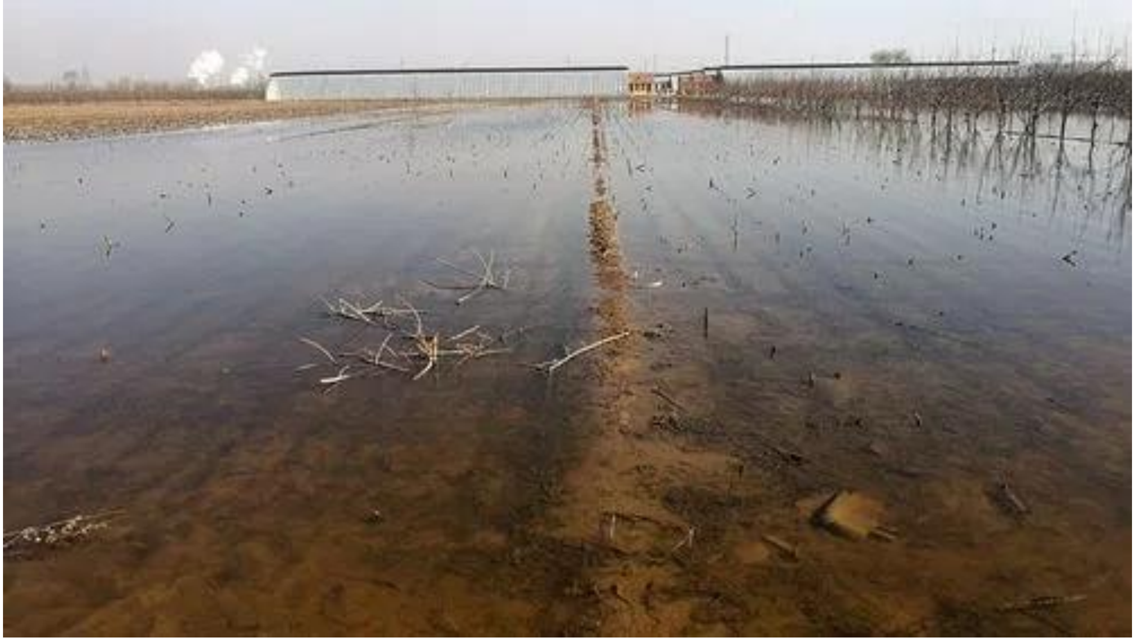

Figure S1. Flood irrigation 1.

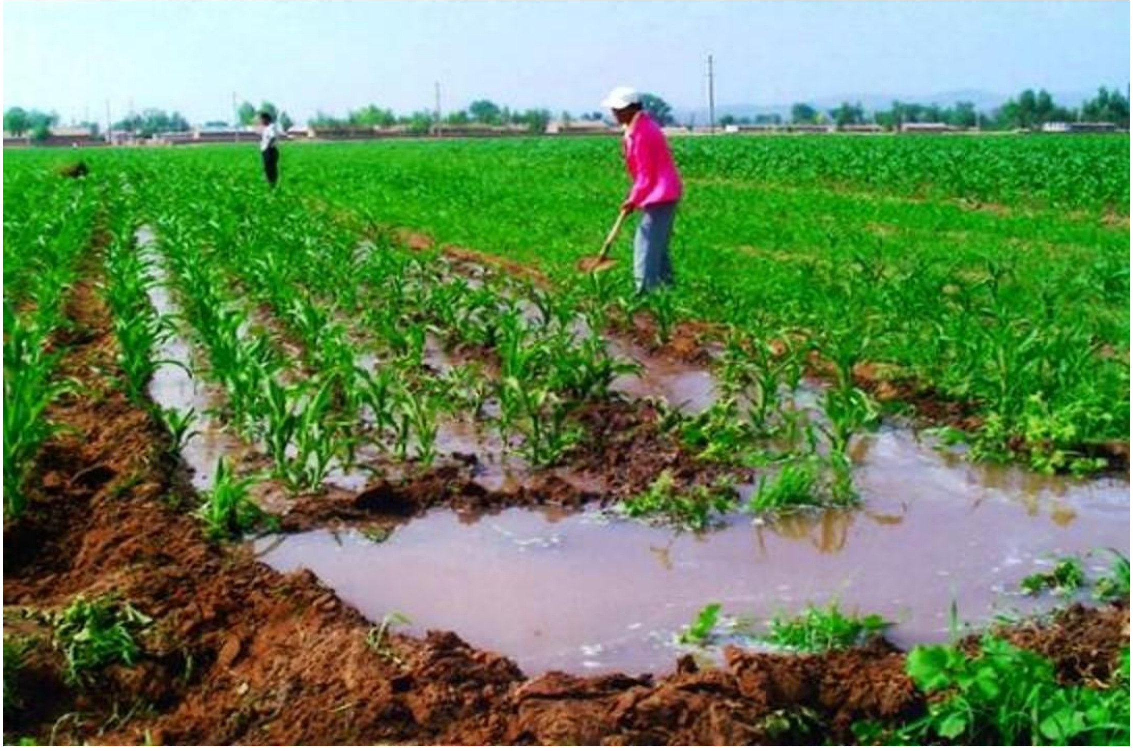

Figure S2. Flood irrigation 2.

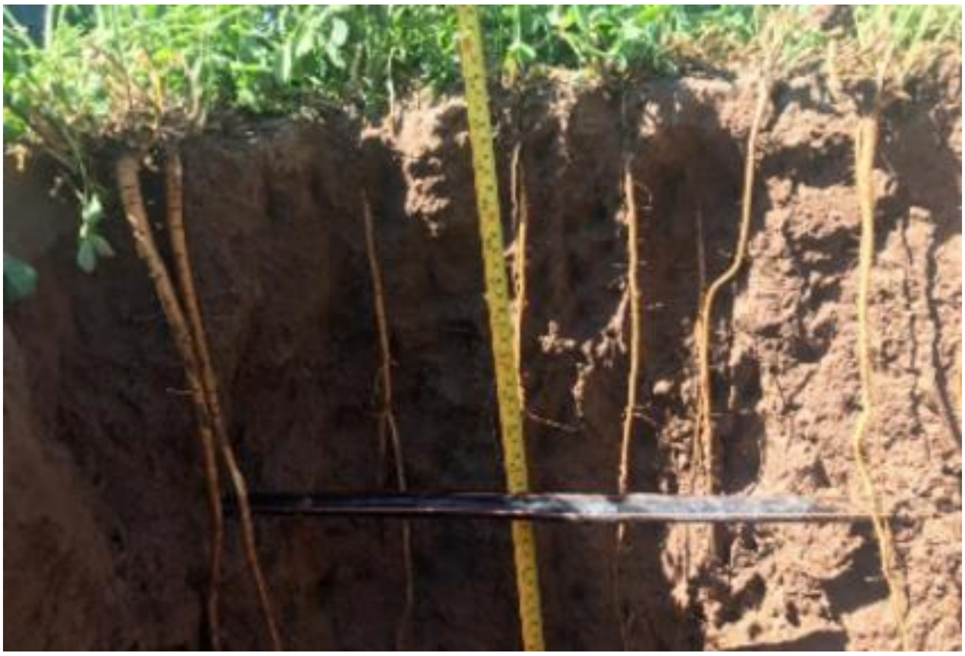

Figure S3. Root depth of *Alfalfa*.

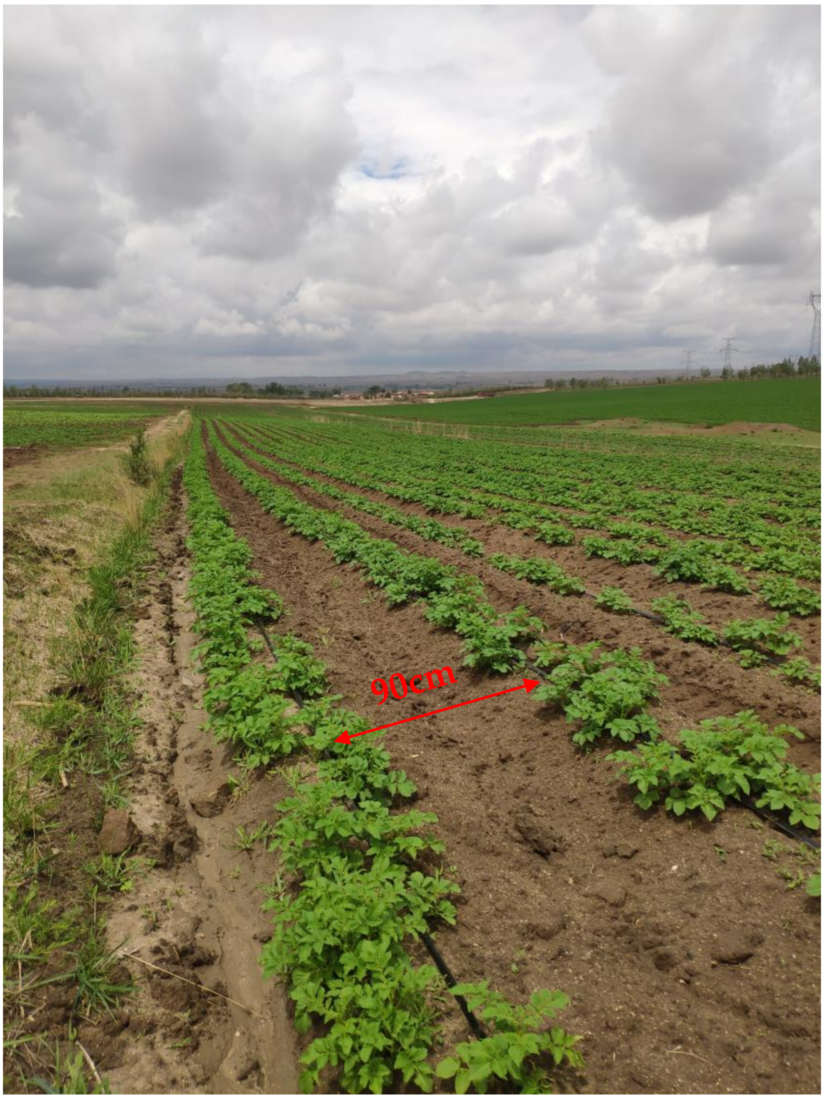

Figure S4. Spacing between potato rows.

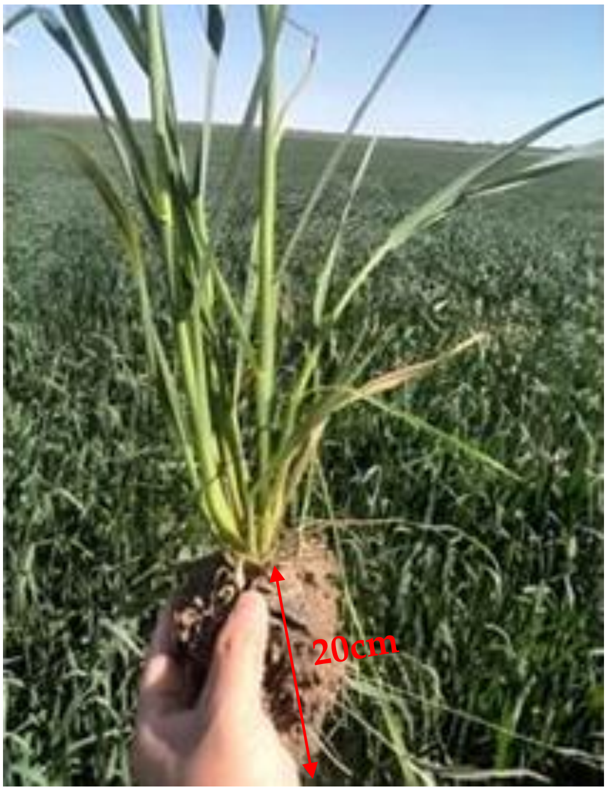

Figure S5. Root depth of oats.

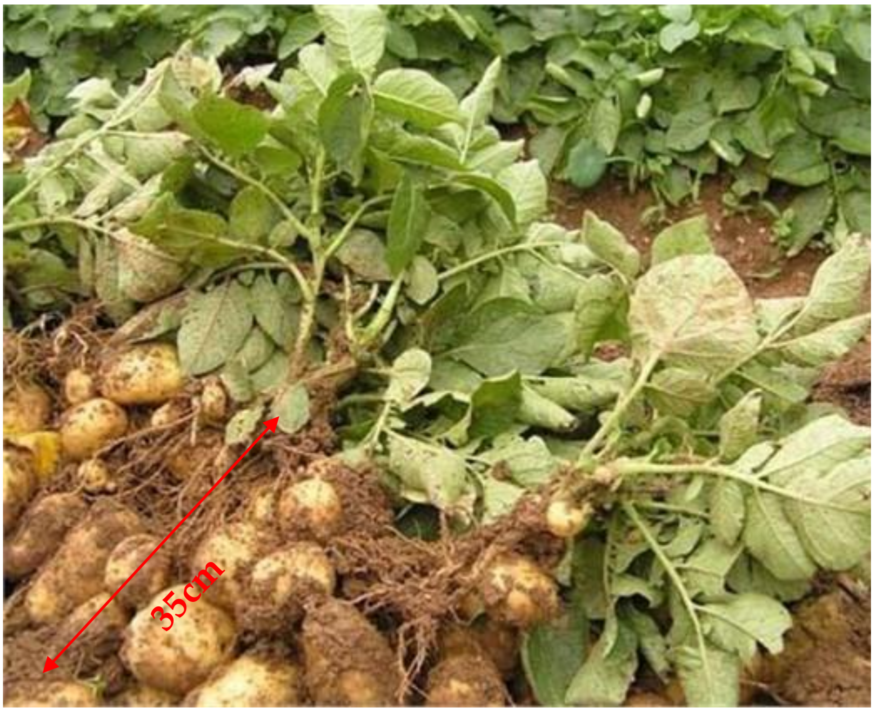

Figure S6. Root depth of potato.

References

1. Osborne, S.L.; Chim, B.K.; Riedell, W.E.; Schumacher, T.E. Root Length Density of Cereal and Grain Legume Crops Grown in Diverse Rotations. *Crop. Sci.* **2020**, *60*, 2611–2620.
2. Boguszewska-Mańkowska, D.; Zarzyńska, K.; Nosalewicz, A. Drought Differentially Affects Root System Size and Architecture of Potato Cultivars with Differing Drought Tolerance. *Am. J. Potato Res.* **2020**, *97*, 54–62.
